# Rapid *In Vitro* Derivation of Endothelium Directly From Human Cancer Cells

**DOI:** 10.1371/journal.pone.0077675

**Published:** 2013-10-09

**Authors:** Jennifer D. Elster, Terence F. McGuire, Jie Lu, Edward V. Prochownik

**Affiliations:** 1 Division of Hematology/Oncology, Department of Pediatrics, Children’s Hospital of Pittsburgh of UPMC, Pittsburgh, Pennsylvania, United States of America; 2 Department of Microbiology and Molecular Genetics, the University of Pittsburgh School of Medicine, Pittsburgh, Pennsylvania, United States of America; 3 The University of Pittsburgh Cancer Institute, Pittsburgh, Pennsylvania, United States of America; Massachusetts General Hospital/Harvard Medical School, United States of America

## Abstract

The development of an independent blood supply by a tumor is essential for maintaining growth beyond a certain limited size and for providing a portal for metastatic dissemination. Host-derived endothelial cells (ECs) residing in and compromising the tumor vasculature originate via distinct processes known as sprouting angiogenesis and vasculogenesis. More recently ECs originating directly from the tumor cells themselves have been described although the basis for this phenomenon remains poorly understood. Here we describe *in vitro* conditions that allow lung and ovarian cancer cells to undergo a rapid and efficient transition into ECs that are indistinguishable from those obtained *in vivo*. A variety of methods were used to establish that the acquired phenotypes and behaviors of these tumor-derived ECs (TDECs) closely resemble those of authentic ECs. Xenografts arising from co-inoculated *in vitro*-derived TDECs and tumor cells were also more highly vascularized than control tumors; moreover, their blood vessels were on average larger and frequently contained admixtures of host-derived ECs and TDECs derived from the initial inoculum. These results demonstrate that cancer cells can be manipulated under well-defined *in vitro* conditions to initiate a tumor cell-to-EC transition that is largely cell-autonomous, highly efficient and closely mimics the *in vivo* process. These studies provide a suitable means by which to identify and perhaps modify the earliest steps in TDEC generation.

## Introduction

At its earliest stages, an incipient tumor fulfills its metabolic needs through simple diffusion of nutrients and waste products [1-3]. Upon reaching a certain critical mass, however, diffusion no longer suffices for this purpose and further growth requires the development of an independent vasculature [3,4]. Without this, tumor dormancy ensues and may persist for years during which time additional tumor cell proliferation is balanced by apoptotic or necrotic death [5,6]. The induction of an “angiogenic switch”, whereby a vascular supply is no longer rate-limiting, is now recognized as a critical determinant of a tumor’s subsequent growth, its communication with the systemic circulation, and its metastatic dissemination [3,4]. Consistent with these findings, vascular density is a well-recognized prognostic factor in many types of cancer including breast cancer, neuroblastoma, and astrocytoma/glioblastoma [7-10]. 

The angiogenic switch is a complex process that involves the elaboration by the avascular tumor of cytokines and growth factors including vascular endothelial growth factor (VEGF), fibroblast growth factor (bFGF), platelet-derived growth factor (PDGF), transforming growth factor beta (TGF-β), and a variety of angiopoietins [1,11-13]. Some of these are chemo-attractants that mobilize both mature and progenitor endothelial cells (ECs) from the bone marrow and drive their maturation and organization into blood vessels (“vasculogenesis”), whereas others induce the endothelium of adjacent blood vessels to proliferate and invade the tumor (“sprouting angiogenesis”) [14-16]. 

The extra-tumoral origin of the neovasculature implies that its component cells are both genetically normal and stable and thus largely immune to developing the chemotherapeutic resistance that commonly arises within the genomically unstable tumor cell population. Indeed, anti-angiogenesis therapies are partly predicated on the assumption that the tumor vasculature retains the genomic stability of its precursor cell population [17,18]. Bevacizumab, the first clinically useful angiogenesis inhibitor, is a humanized anti-VEGF monoclonal antibody (mAb) that showed early promise in treating a variety of advanced cancers [19-22]. However, virtually all responses are incomplete and/or transient as tumors eventually re-vascularize and become unresponsive to further treatment with the mAb. As a result, overall patient survival has been improved only modestly, if at all [19-23].

Recently, we and others have provided a potential explanation for the incomplete responses to anti-angiogenesis agents by showing that a significant sub-population of tumor-associated ECs derive directly from the tumor cells themselves [24-28]. These “tumor-derived ECs” (TDECs) express a variety of EC markers, down-regulate epithelial markers and form functional vessels *in vivo* where they admix with extra-tumorally-derived ECs. Because they contain the same marker chromosomes as the tumor cell population, it was suggested that, like the tumor cells themselves, TDECs were genomically unstable [24-28]. Consistent with this idea, the serial passage of TDECs leads to the eventual emergence of clonally-derived populations that express progressively more robust EC phenotypes and are genetically related to but distinct from both tumor cells and early-passage TDECs [24]. TDEC’s have been identified in a murine model of glioblastoma [27] and in human glioblastoma xenografts [26,28]. Earlier but inconclusive studies had also suggested the presence of TDECs in other primary human tumors [29-31]. These findings suggest that TDEC generation is a widespread, if not universal, phenomenon and that resistance to anti-angiogenic therapies may emerge as a result of inherent TDEC genomic instability. 

The finding that TDECs constitute a functionally significant and distinct EC population raises a number of questions that are difficult or impossible to address by studying primary tumors or tumor xenografts. These include the nature and relative importance of signals that initiate the tumor cell to TDEC transition, the time frame over which this occurs, whether TDEC development and maintenance are cell autonomous and whether all cells within a tumor are capable of generating TDECs. We describe here the development of an *in vitro* system that allows us to address these questions. Using conditions that favor the growth of ECs and mimic the hypoxic and nutrient-deprived tumor microenvironment, we show that a robust EC phenotype can be readily generated from tumor cells and that optimal induction requires synergistic cooperation of these factors. The properties of *in vitro-*derived TDECs are virtually indistinguishable from those isolated directly from tumors. Moreover, their co-inoculation with tumor cells leads to the development of xenografts with a denser tumor vasculature and, in some cases, a more rapid growth rate. These studies thus provide a simple and quantitative means by which TDEC ontogeny can be studied and manipulated from its inception under defined conditions.

## Results

### Expression of EC markers in human tumor cells under defined *in vitro* conditions

We initially sought to identify conditions that promote a tumor cell to TDEC transition *in vitro*. For these studies, we utilized the human H460 and CaLu1 non-small cell lung cancer, the PC3 prostate cancer and the OVCAR3 ovarian cancer cell lines. In choosing culture conditions, we hypothesized that a combination of EC-specific growth medium and moderate hypoxia, perhaps coupled with the depletion of certain nutrients, could recapitulate the *in vivo* environment that provides the signal(s) for TDEC initiation. Tumor cells were therefore cultured in either EC-specific EGM-2 medium + normoxia (condition 1), standard growth medium + hypoxia (condition 2) or a combination of EGM-2 medium + hypoxia (condition 3). For OVCAR3 cells, we also used Glutamax medium supplemented with EC specific factors identical to those in EGM2 medium but deprived of asparagine, aspartic acid, glutamine and proline + hypoxia (condition 4) or the same medium + normoxia (condition 5). We refer to this set of conditions collectively as “EC-promoting”. Tumor cells maintained in their standard recommended growth medium under normoxic conditions served as starting point controls. Cell lysates were prepared from these samples and analyzed by immunoblotting for the expression of von Willebrand’s factor (vWF), which is reliably induced during the tumor cell to TDEC transition *in vivo* [24,25]. As shown in Figure S1, most of the tumor cell lines grown under standard conditions showed little or no expression of vWF. In contrast, vWF was induced to varying degrees under different EC-promoting conditions with the highest and most sustained levels usually being seen under condition 3. Although the time to achieve maximal induction varied among the tested lines and was sometimes transient, it was generally maximal between d 3-5 and did not subsequently increase. 

**Figure 1:**
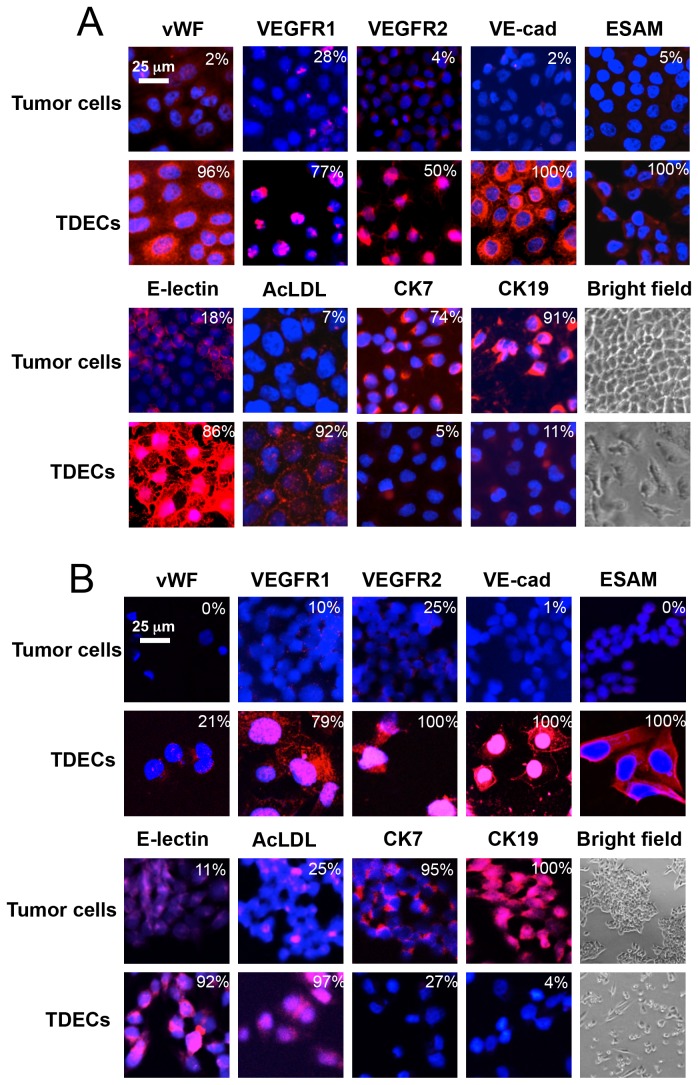
Immunofluorescent staining of tumor cells grown under standard conditions and under optimal conditions to induce TDECs. (*A*) H460 cells were grown for 5 d under condition 3. (*B*) OVCAR3 cells were grown for 5 d under condition 4. Antibody staining for the EC-specific markers vWF, VEGFR1, VEGFR2, VE-cadhherin, ESAM, binding of E-lectin and uptake of acetylated AcLDL were performed on both sets of cells as previously described [24,25]. Epithelial marker staining was performed for CK7 and CK19. Counterstaining with DAPI was performed to visualize nuclei. Bright field images of tumor cells and TDECs are also included. Images were obtained at either 40-60X magnification (confocal) or 10X magnification (bright field). Numbers in the upper right hand corner of each panel indicate the percentage of each population that demonstrated any evidence of staining, irrespective of its intensity. Similar results were obtained in at least three independent experiments. Scale bar = 25 um.

 Having established conditions under which to achieve maximal vWF expression in each cell line, we expanded our initial analyses to include additional EC-specific markers using immuno-fluorescence-based assays, as previously described [24,25]. For these studies, we concentrated on H460 and OVCAR3 cells cultured for 5 d under conditions 3 and 4, respectively. The EC-specific proteins analyzed again included vWF as well as VEGFR1, VEGFR2, VE-cadherin and EC-selective adhesion molecule (ESAM). In addition, binding of *Ulex europeus* lectin (E-lectin), uptake of acetylated low-density lipoprotein (AcLDL), and morphology were followed as indicators of more complex EC phenotypes. Cytokeratins 7 (CK7) and 19 (CK19) were also examined as markers for the epithelial phenotype. As shown in Figure 1, all EC markers were induced in both cell lines whereas both cytokeratins were markedly down-regulated. These changes involved both the percentage of cells that stained for the markers as well as the intensity of positive cell staining. Thus, the expression patterns of all markers closely mimicked those seen with TDECs derived from actual tumor xenografts [24,25].

### Functional analysis of *in vitro*-derived TDECs

**Figure 2:**
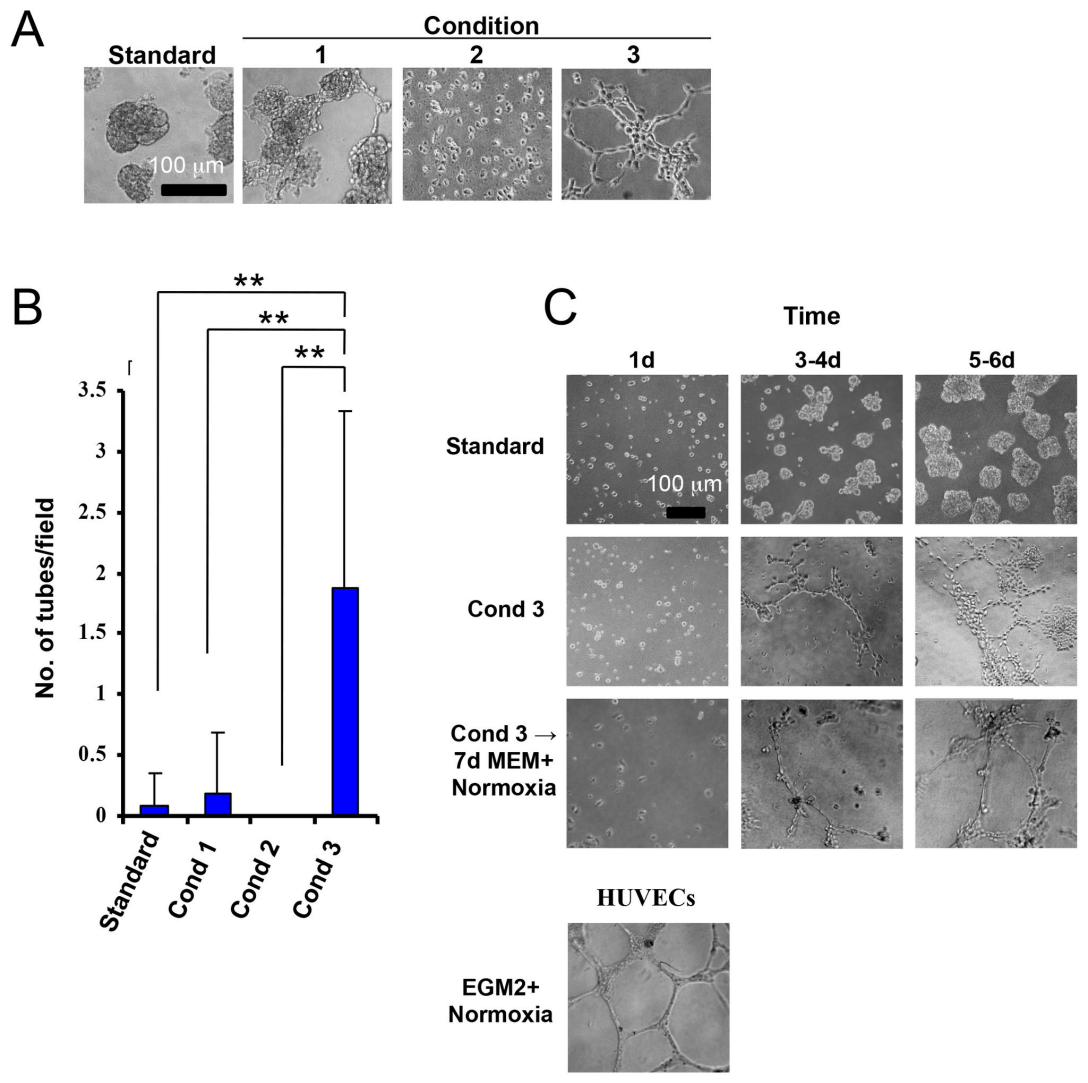
Tube formation by TDECs. (*A*) H460 tumor cells were induced to form TDECs by 5 d of exposure to the indicated conditions. 2 x 10^4^ cells from each group were then plated on Matrigel in a tube formation assay under normoxic conditions for 5 d, as previously described [24,25]. Untreated H460 cells grown under standard conditions served as controls. Typical light microscopic fields are shown. All photos are shown at identical magnifications. (*B*) The results shown in (*A*) are graphically depicted as the mean number of completely enclosed tubes per field ± SEM. p values were obtained using one-way ANOVA (**: p < 0.01). (*C*) H460 tumor cells were grown under standard conditions or condition 3 for the indicated times. Half the cells were then immediately plated on Matrigel and grown under normoxic conditions for an additional 6 d. The remainder of the cells were returned to standard conditions for 7 d prior to being plated in Matrigel for 6 d to assess the persistence of tube-forming potential. HUVECs were used as a positive control for tube formation. Brightfield photographs were taken at 10X magnification. Similar results were obtained in four independent experiments (A) and two independent experiments (C). Scale bar = 100 um.

To compare further the EC phenotypes of *in vitro-*derived TDECs with those from actual tumor xenografts, we examined the former cells for their ability to form tube-like structures in semi-solid medium. For these studies, H460 cells were cultured under standard growth conditions (control) or under conditions 1-3 for 5 d at which time they were plated on semi-solid medium and cultured under normoxia for 5 additional days. Under both standard and EC-promoting conditions 1 or 2, H460 cells persisted as individual cells or formed amorphic acini whereas culturing under condition 3 greatly enhanced tube formation (Figure 2A). Quantification of this showed tube formation by H460 cells to be increased by 10- to >50-fold over that seen under conditions 1 or 2 and by nearly 25-fold over that of standard conditions (Figure 2B). Although the numbers of tubes formed and their quality were inferior to those originating from human umbilical vein ECs (HUVECs) (Figure 2C), they were indistinguishable from the tubes formed by *in vivo*-derived TDECs [24,25]. These observations indicate that H460-derived TDECs could form tubes in the normoxic conditions under which the tube-formation assay was conducted and suggested that the TDEC phenotype might be stable. To test this, H460 cells, first exposed to condition 3, were plated directly into semi-sold medium as described for Figure 2A or were maintained under standard conditions for an additional 7 d before plating in a tube formation assay. Surprisingly, under this latter regimen, the tube forming ability of TDECs was not only maintained but the resultant tubes more closely resembled those formed by HUVECs (Figure 2C). Thus, while TDEC tube-forming potential was shown to require hypoxia, it was retained for at least 2 weeks following a return to standard growth conditions.

### A biosenser-based assay to monitor *in vitro* TDEC induction

**Figure 3:**
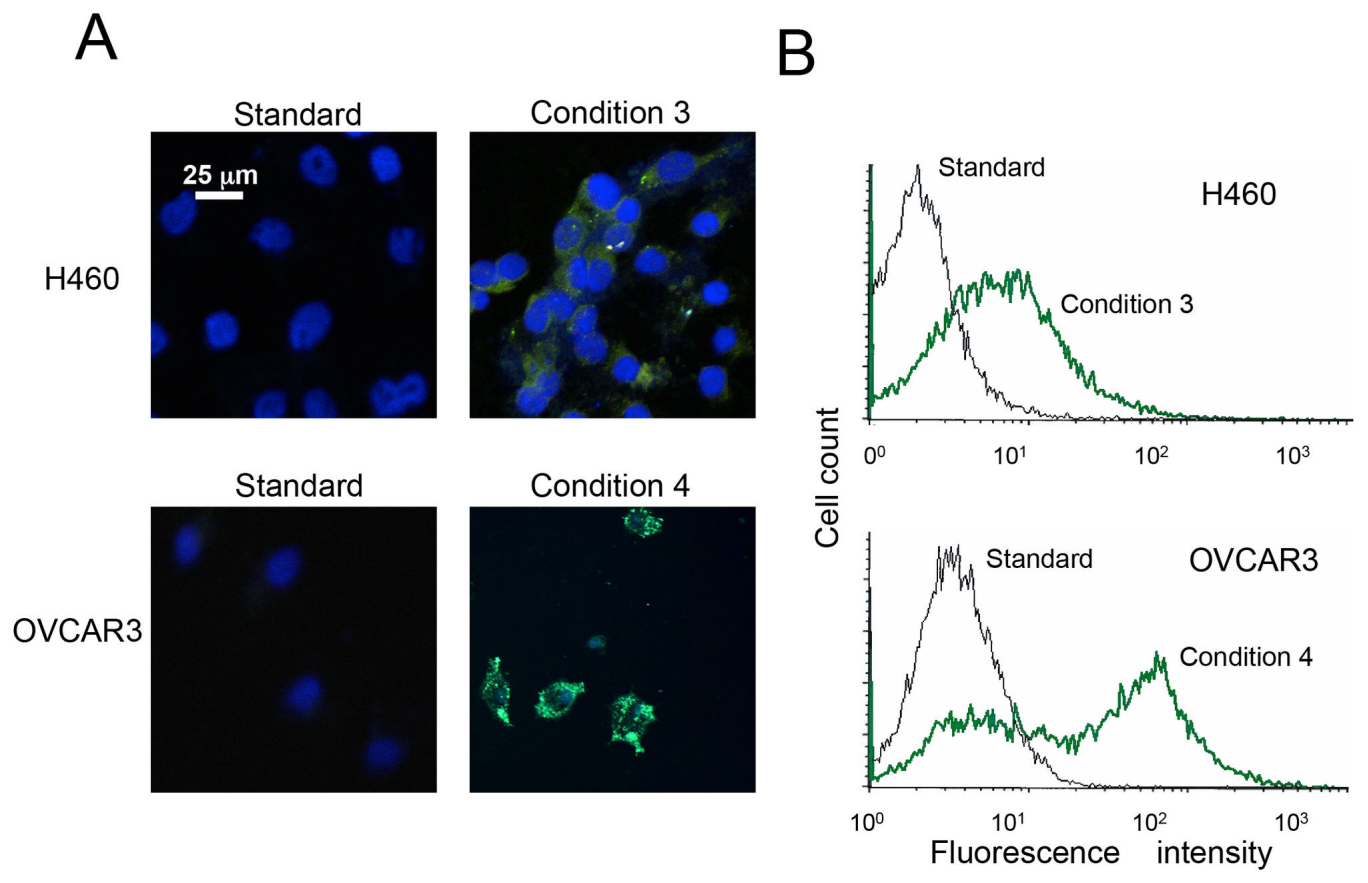
Hypoxia, EC-specific growth medium, and nutrient deprivation induce Tie2-dependent EGFP expression in tumor cells. Separate cultures of H460 and OVCAR3 tumor cells were stably co-transfected with Tie2-EGFP plasmid and pFR400 encoding a mutant form of dihydrofolate reductase [36,37] and selected in G-418 and increasing concentrations of methotrexate to allow amplification of the two tandemly integrated vectors and a corresponding increase EGFP signal intensity. Cells were exposed to conditions shown previously to induce the maximal TDEC phenotype (i.e., condition 3 for H460’s and condition 4 for OVCAR3) and compared to control cells grown under standard conditions. (*A*) Fluorescence micrographs of each cell line after five days of growth under each set of conditions. (*B*) Flow cytometric analysis of the same cells. Representative results of at least three independent experiments are depicted. Scale bar = 25 um.

To confirm and better quantify the *in vitro* tumor cellTDEC transition, we generated separate populations of H460 and OVCAR3 cells stably expressing enhanced green fluorescent protein (EGFP) under the control of the EC-specific angiopoietin receptor (Tie2) promoter [32]. Under standard growth conditions, these cells expressed little EGFP, even when assessed by flow cytometry (Figure 3). Following TDEC induction under condition 3 for H460 cells and condition 4 for OVCAR3 cells, 3-5-fold increases in the EGFP signal were routinely observed (Figure 3). Thus, Tie2-EGFP induction serves as a simple, reproducible and quantifiable surrogate assay for TDEC differentiation that, in live cells, accurately mirrors the results obtained by more standard assays of EC phenotype. It also provides direct evidence for transcriptional up-regulation of at least some of the markers during the course of TDEC differentiation and helps to substantiate their coordinate up-regulation over a 4-5 day time-frame (Figure S2). 

The foregoing results, together with those depicted in Figure 2, suggest that a majority of tumor cells display sufficient plasticity to allow their *in vitro* transition into TDECs. To test this directly, we examined 80 single cell clones derived from H460 and OVCAR3 cells to determine the degree to which they could express EC markers. As shown in Table 1, virtually every clone showed high-level uptake of AcLDL, E-lectin binding and Tie2-driven EGFP expression under EC-promoting conditions whereas only faint expression of these markers could be detected in cells maintained under standard conditions. Thus, as previously suggested for *in vivo-*derived TDECs [25], the acquisition of the EC phenotype is not confined to any particular cellular subset.

**Table 1:** Endothelial differentiation of human tumor line single cell clones.

	H460					OVCAR-3			
	% + cells (Std Condition/Condition 3)					% + cells (Std Condition/Condition 4)			
Clone No.	E-Lectin	AcLDL	Clone No.	Tie-2-GFP	Clone No.	E-Lectin	AcLDL	Clone No.	Tie-2-GFP
1	<3/90-95	<3/>95	21	<3/90-95	41	<3/80	<3/90	61	<3/>95
2	<3/90-95	<3/>95	22	<5/90-95	42	<3/90	<3/90	62	<3/>95
3	5/80	<3/90-95	23	<5/>95	43	<3/>95	<3/95	63	<3/<3
4	5/>95	<3/>95	24	<3/>95	44	<3/>95	<3/90	64	<3/>95
5	5/>95	<3/80	25	3-5/>95	45	<3/>95	<3/90	65	<3/>95
6	5/>95	<3/90-95	26	<3/>95	46	<3/90	<3/95	66	<3/>95
7	5/90-95	<3/>95	27	<3/<3	47	<3/80	<3/95	67	<3/>95
8	5/>95	5/90-95	28	<3/>95	48	<3/90	<3/90	68	<3/<3
9	5/>95	5/>95	29	<3/90-95	49	<3/90	<3/90	69	<3/>95
10	5/80-85	5/>95	30	<3/>95	50	<3/50	<3/90	70	<3/<3
11	10/>95	5/90-95	31	<3/>95	51	<3/>95	<3/95	71	<3/>95
12	<3/>95	<3/>95	32	<3/<3	52	<3/<3	<3/50	72	<3/>95
13	5/>95	<3/>95	33	<3/>95	53	<3/30-40	<3/95	73	<3/>95
14	10/>95	3/>95	34	5/>95	54	<3/30-40	<3/80	74	<3/<3
15	10/90-95	<3/>95	35	<3/>95	55	5/90-95	<3/95	75	<3/<3
16	<3/90-95	3/>95	36	<3/>95	56	5/>95	<3/95	76	<3/>95
17	<3/90-95	<3/>95	37	<3/>95	57	5/>95	<3/95	77	<3/>95
18	5/>95	3/>95	38	<3/<3	58	5/90-95	<3/95	78	<3/>95
19	5/>95	<3/>95	39	<3/<3	59	5/>95	<3/95	79	<3/<3
20	<3/>95	<3/90-95	40	5/>95	60	<3/>95	<3/95	80	<3/>95

Cell sorting was used to seed individual H460 and OVCAR3 cells into 96-well plates. 20 clones derived from each cell type were expanded and evaluated under standard or EC-promoting conditions for E-lectin binding and AcLDL uptake. An additional 20 clones each derived from H460-Tie2-GFP and OVCAR3-Tie2-GFP cells were also evaluated for the expression of GFP. The percent of positive cells for each of these EC-specific markers following 5 days of propagation were measured under standard conditions or conditions 3 or 4 and the two values obtained are separated by the “/”. These studies do not take into account the large differences in marker intensity that occurred following hypoxic propagation, which are illustrated in Figure S2.

### TDECs incorporate into the tumor neovasculature, enhance tumor growth and increase vessel density

**Figure 4:**
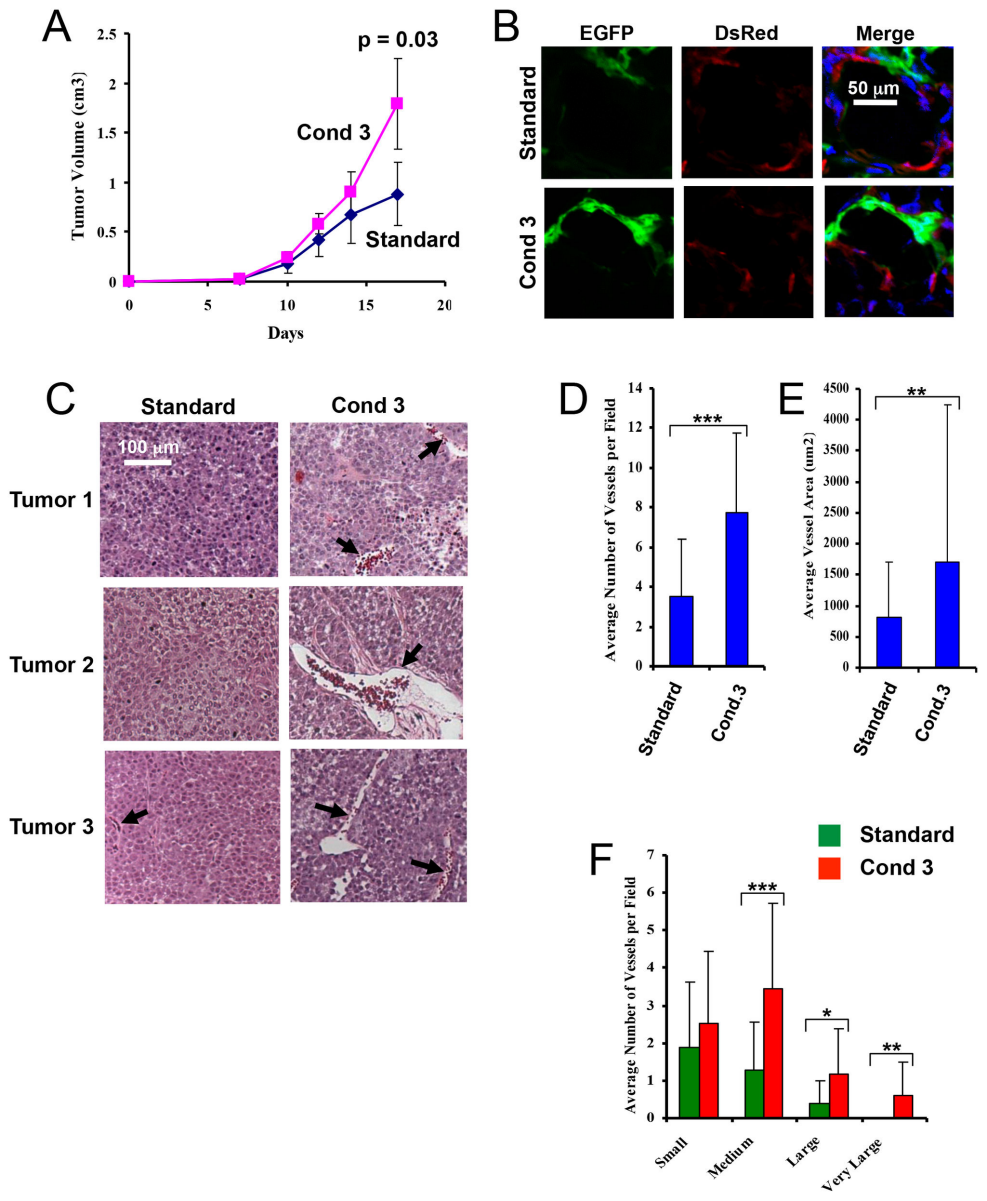
TDECs accelerate H460 tumor xenograft growth and increase tumor vessel density and size. EGFP-tagged H460 cells were maintained for 5 d under condition 3. The resulting TDECs were then mixed with a 20-fold excess of DsRed-tagged H460 tumor cells grown under standard conditions and a total of 10^6^ cells were inoculated subcutaneously into the flanks of nude mice and propagated as tumor xenografts. Control tumors consisted of the same mix of DsRed-tagged tumor cells and EGFP-tagged tumor cells propagated under standard conditions. (*A*) Graphical representation of tumor growth. Tumor volumes were determined at the indicated times and the averages were plotted (± SEM). The *p* value shown for day 17 tumor volumes was derived using a one-tailed Student’s t-test. (*B*) Confocal fluorescence images of frozen sections tumors from each of the two groups. Scale bar = 25 um. (*C*) Representative low-power hematoxylin-eosin-stained paraffin-embedded tissue sections taken from typical tumors in each of the two groups. (*D*) Graphical depiction of the mean number of tumor blood vessels per field (±SEM) in typical fields of each tumor type. The total number of fields examined was 32 for condition 3 tumors and 24 for standard condition tumors. Only vessels exhibiting distinct lumens and containing red blood cells, indicated by *black* *arrows*, were counted. (*E*) Graphical depiction of the mean blood vessel cross-sectional area (±SEM) in the two tumor types. The total number of vessels measured was 85 from standard condition tumors and 248 from condition 3 tumors. (*F*) The mean number of tumor blood vessels with cross-sectional areas (±SEM) that were small (30-499 um^2^), medium (500-1999 um^2^), large (2000-4999 um^2^), and very large (≥ 5000 um^2^), as measured using ImageJ software. Statistical analysis was performed using a one-tailed Student’s *t* test (*, *p* < 0.01; **, *p* < 0.001; ***, *p* < 0.0001). Similar results were obtained in two independent experiments.

Our previous findings that *in vivo-*derived TDECs can incorporate into the tumor neo-vasculature and increase blood vessel density [24,25] suggested that *in vitro*-derived TDECs might behave similarly. To address this, EGFP-tagged H460 tumor cells [25] were cultured under condition 3 for 5 d, mixed with a 20-fold excess of *Discosoma* *sp.* red fluorescent protein (DsRed)-tagged H460 tumor cells and propagated as subcutaneous xenografts in immunocompromised mice. Control injections were performed identically but with the EGFP+ population grown under standard conditions. Tumors arising from the former cell mixture grew significantly more rapidly than those from the latter (Figure 4A). Fluorescence microscopy of frozen sections also showed the blood vessels of the former tumors to be enriched for EGFP+ ECs (Figure 4B), indicating a predilection for *in vitro*-derived TDECs to incorporate into the tumor vasculature. Finally, the blood vessels of the former tumors were both denser and larger than those of the latter tumors (Figure 4C-4F). Similar experiments performed with OVCAR3 cells showed that the resultant tumor xenografts, while not growing significantly faster than their control counterparts, did contain greater proportion of EGFP+ luminal TDECs and a denser vasculature (Figure S3). Thus, the co-injection of *in vitro-*derived TDECs facilitates tumor neovascularization, which, in some cases, allows for accelerated tumor growth.

## Discussion

Recent observations in a variety of different cancers have indicated that the ECs comprising the tumor neo-vasculature can originate directly from the tumor cells themselves and co-exist with ECs derived from extra-tumoral sources [24-28]. Much like their tumor cell predecessors, these TDECs are genomically unstable [24]. This may confer a survival advantage in the face of anti-angiogenesis therapies, thus perhaps accounting for the transitory effects of these agents [19-22]. Indeed, we have observed that *in vitro-*derived TDECs such as those described here can be derived in EGM2 medium lacking VEGF and thus appear to be independent of this growth factor even in the absence of prior selection. Moreover, the extremely low levels of VEGF transcripts that were detected in H460 and OVCAR3 cells by real time qRT-PCR, were not significantly changed following induction of the TDEC phenotype (not shown).

Our findings indicate that tumor cells can be manipulated *in vitro* under defined conditions to generate TDECs that are both biochemically and functionally similar to those originating *in vivo* [24,25]. That virtually all tumor cells can acquire EC-like properties (Table 1) while losing their epithelial characteristics is consistent with our previous finding that single-cell clones derived from tumor cells can generate TDECs *in vivo* [25]. Thus, the capacity for TDEC generation appears to be a common, if not universal, trait that is shared by a majority of the tumor cell population and is cell autonomous. The variable proportion of TDECs found in different tumors [25] may thus be less indicative of any inherent generational capacity than of competing processes such as sprouting angiogenesis and vasculogenesis, sub-optimal conditions for TDEC induction and lack of other selective pressures such as prior therapeutic interventions. The property appears to be confined to tumor cell populations as we have not observed ECs to arise from non-transformed cells such as human embryonic kidney or primary or immortalized bronchial and mammary gland epithelial cells, when cultured under conditions identical to those described herein (not shown).

The ability to generate TDECs under defined *in vitro* conditions answers several questions that cannot be readily addressed using *in vivo* models where the tumor microenvironment is often heterogenous, subject to rapid change [1,33] and where TDEC formation is likely to compete with additional vascular remodeling processes. These questions include the nature and interrelationships of the inductive signals for TDECs and their timing. Obvious candidates for such signaling molecules include EC-specific growth factors such as VEGF, bFGF and TGF-beta, as well as hypoxia, all of which are strong inducers of tumor neovascularization [3-5,11-14]. Our results indicate that, at least *in vitro*, neither the growth factors supplied by EC-specific growth medium nor hypoxia alone are particularly potent inducers of the tumor cell to TDEC transition but that, in combination, they are highly synergistic. This is not entirely unexpected as these factors have been long known to be necessary for the generation and maintenance of the tumor neovasculature arising from more traditional sources [34,35]. In some cases, as exemplified by our results with OVCAR3 cells, other factors, such as selective nutrient deprivation or acidosis may play additional supportive roles and remain to be explored more thoroughly. The precise nature of these effectors and their relative importance for TDEC generation are likely to be quite dependent on inherent properties of specific tumor types, acquired secondary changes and various other competing and cooperating environmental factors. It is also likely that the length of exposure to various inductive factors as well as when during the tumor cell to TDEC transition period they are operative will play important roles. All of these questions should be addressable using the types of assays described here, which allow for the precise control and timing of potential environmental cues.

Our previous studies had indicated that both the expression of EC-specific markers and the function of *in vivo*-derived TDECs were cell line-dependent and this was borne out by the current studies. Perhaps the most obvious example of this was illustrated by the differences between H460 and OVCAR3 TDECs with regard to their ability to affect tumor xenograft growth rates and vessel size (Figures 4 and S3). Whether this reflects intrinsic functional differences of TDECs, the growth patterns of the tumor cells themselves, stromal interactions or a combination of these factors is currently unknown. Definitive evidence for such cause-effect relationships awaits future confirmation.

The ability to recapitulate the tumor cell to TDEC transition efficiently, rapidly and under well-defined and easily alterable conditions should now permit more thorough evaluations of the precise roles played by individual inductive factors in facilitating this process as well as their relative importance. The ease with which TDECs can be generated also suggests that they may serve as valuable reagents to be used in the identification of novel agents that prevent this process.

## Materials and Methods

### Ethics Statement

All mouse studies were conducted according to Animal Welfare Act and the Public Health Service Policy and approved by the University of Pittsburgh’s Institutional Animal Care and Use Committee (IACUC) (Permit Number: 0812276). Animals were housed in pathogen-free units at Children’s Hospital of Pittsburgh in compliance with IACUC regulations.

### Animals

Age and gender-matched nu/nu mice were purchased from Harlan Sprague-Dawley Laboratories (Indianapolis, IN). Tumor xenograft studies were conducted as previously described [24,25].

### *In vitro* induction of TDECs and tumor xenograft growth

NCI-H460 human lung carcinoma (H460), CaLu-1 human lung carcinoma, PC3 prostate cancer, and OVCAR3 ovarian cancer cell lines were obtained from the American Type Culture Collection (Manassas, VA) and were maintained under normoxic “standard” conditions as previously described [24,25]. Culture media included alpha Minimal Essential Medium (“MEM”) for H460 and PC3 and McCoy’s 5a medium for CaLu-1 and OVCAR3, both supplemented with 10% fetal bovine serum, 2 mM glutamine, 110 ug/ml pyruvate, minimum non-essential amino acids, 100 ug/ml streptomycin and 100 units/ml penicillin G). Human umbilical vein endothelial cells (HUVECs) and EC-specific EGM-2 growth medium were purchased from Cambrex Bio Science (Walkerville, MD). All cell lines were maintained in a 5% CO_2_ atmosphere at 37C. For most tumor lines, induction of an EC phenotype was achieved by culturing cells under a variety of conditions. These included EGM-2 medium under normoxic conditions (condition 1); standard medium under hypoxic condition [1% O_2_ in a Hypoxic Glove Box incubator (Coy Laboratory Products Inc., Grass Lake, MI)] (condition 2); or EGM2 medium + hypoxia (condition 3). For some experiments, optimal induction additionally required nutrient-deficient medium [Glutamax D-MEM medium lacking the non-essential amino acids asparagine, aspartic acid, glutamine, and proline (Invitrogen, Inc.)] under hypoxic conditions but otherwise containing the identical growth factors and supplements as EGM-2 medium (condition 4). A final condition included the above nutrient-deficient medium plus normoxia (condition 5). Lentiviral packaging and infections to produce EGFP- or DsRed-tagged cells were performed as previously described [25]. 

Subcutaneous tumor xenografts were obtained by inoculating 10^6^ tumor cells, which were comprised of EGFP-tagged TDECs and DsRed-tagged tumor cells (1:20), subcutaneously into the flanks of nu/nu mice as previously described [25]. Tumor volume measurements were made every 2–3 days until the maximal allowable diameter of ca. 2 cm was reached (typically 4-6 wks for both H460 and OVCAR3 cells) at which point the tumors were excised. Separate fragments of tumors were used for the preparation of frozen and paraffin-embedded sections. For evaluation of tumor blood vessels, frozen sections were fixed 10 min with 4% paraformaldehyde and stained with DAPI, as previously described [25]. Fluorescence microscopic images of EGFP- and DsRed-labeled vessels were obtained using an Olympus Fluoview 1000 confocal microscope at a magnification of 40-60X. For vessel counting, hematoxylin-eosin staining of paraffin-embedded sections from tumor xenografts was performed by the University of Pittsburgh Core Histology Laboratory. ImageJ software was used to calculate blood vessel density.

### Evaluation of EC-specific markers and functions

For immunoblotting cell monolayers were washed with PBS and lysed with an appropriate volume of SDS-PAGE sample buffer. Equivalent amounts of protein were loaded onto polyacrylamide gels, transferred to nitrocellulose, and immuno-blotted with an anti-human von Willebrands factor (vWF) antibody as previously described [25]. After incubation with an appropriate horseradish peroxidase-conjugated secondary antibody, immunoblots were developed using an enhanced chemiluminescence system (Pierce Biotechnology, Rockford, IL).

For immuno-staining, cells were grown on glass coverslips, fixed in PBS-4% paraformaldehyde and stained with antibodies against the endothelial cell markers vWF, VEGFR1, VEGFR2, VE-cadherin and ESAM as previously described [25]. Alexa 594-conjugated antibodies were used as secondary stains. Additional EC-specific staining was achieved using rhodamine-tagged *Ulex europeus* lectin (E-lectin: Vector Laboratories, Burlingame, CA) and through the uptake of AlexaFluor 594-tagged acetylated low-density lipoprotein (AcLDL) (Invitrogen Molecular Probes) both of which were assessed in live cells [25]. Staining for expression of cytokeratins (CK) 7 and 19 was also performed as a way of monitoring loss of the epithelial phenotype after TDEC induction. For all samples, cells were counterstained with DAPI to visualize nuclei. Images for fluorescence confocal microscopy were obtained as described above. Adobe Photoshop CS2 (version 9.0, Adobe Systems, San Jose, CA) was employed for image analysis. 

### Tube formation assay

Tumor cells or TDECs were assessed for tube-formation activity in Matrigel as previously described [25]. Briefly, 24-well plates initially coated with 100 ul Cultrex BME Growth Factor Reduced PathClear matrix (R&D Systems, Minneapolis, MN). Tumor cells or TDECs (5000 cells/well) or HUVECs (10,000 cells/well) were then seeded in EGM-2 medium for the times and under the conditions indicated in the figures. Tube formation was typically assessed between 2-10 d, at which point photographic images were obtained using a Zeiss Axiovert 135 inverted microscope equipped with a Sony DXC-970MD 3CCD Color Video Camera. Statistical analysis was performed using one-way ANOVA, utilizing the VassarStats website (http://www.vassarstats.net/anova1u.html).

### Tie2-EGFP vector

The murine Tie2 promoter (Gen Bank Accession no. AL772277) was a kind gift from Dr. Luigi Naldini [32]. A 1.91 kb NdeI-HindIII fragment encoding upstream promoter sequences and 67 bp of 5’-untranslated sequence was excised from the parental vector, blunt ended with the Klenow fragment of DNA polymerase I and cloned into the blunt ended AseI-BglII sites of the pEGFP-N3 vector (Clontech, Inc. Mountain View CA) from which the CMV promoter had been removed. Proper orientation of the promoter was confirmed by automatic DNA sequencing. Plasmid DNA (1 ug) was co-transfected into H460 and OVCAR3 cells along with 5 ug of pFR400, a mammalian expression vector encoding a mutant form of dihydrofolate reductase with a low affinity for methotrexate [36]. Transfections were performed using Superfect (Qiagen, Valencia CA) followed by selection in G-418 and subsequent step-wise selection of the G-418-resistant pooled clones in 0.25 uM and 1 uM methotrexate in order to amplify the tandemly integrated plasmids [36,37]. 

### Flow cytometry

H460-Tie2-EGFP and OVCAR3-Tie2-EGFP were either grown in standard conditions or induced for 5 days in the appropriate conditions to induce TDECs, as described above. For some experiments, cells were treated with AcLDL or E-Lectin as described for immunofluorescence experiments. Cells were non-enzymatically dissociated, washed with PBS, and fixed with 2% paraformaldehyde. Untransfected cells from each culture condition were similarly prepared and used as negative controls. Fluorescently labeled cells were analyzed on a FACStar flow cytometer (Becton-Dickinson Biosciences, San Jose, CA). 

### Statistical Analysis

One-way ANOVA statistical analysis [utilizing VassarStats website (http://www.vassarstats.net/anova1u.html)] was performed for the bar graph shown in Figure 2B, since four means were being compared simultaneously for one factor (tube formation). All other statistical analysis performed utilized either the one-tailed or two-tailed Student’s *t* test as appropriate. 

## Supporting Information

Figure S1 **Time course of vWF induction in H460, PC3, OVCAR3 and CaLu1 cells exposed to various conditions.** H460, CaLu-1, PC3, and OVCAR cells cultured under the following conditions: “control”, standard growth medium in normoxia; “condition 1”, EC-specific EGM2 medium in normoxia; “condition 2”, standard growth medium in hypoxia (1% O_2_); “condition 3”, EC-specific EGM2 medium in hypoxia; “condition 4” nutrient-deficient GlutaMax medium in hypoxia; and “condition 5”, nutrient-deficient GlutaMax medium in normoxia. After 1, 3, and 5 days, cells were harvested, whole cell extracts were prepared and equivalent amounts of protein were subjected to immunoblotting for von Willebrands Factor (vWF) and beta-actin, as previously described [24,25].
(TIF)

Figure S2 **Time course of tumor cellTDEC transition.** H460, OVCAR3 or H460-Tie2-EGFP and OVCAR3-Tie2-EGFP cells were subjected to EC-promoting conditions and then assayed periodically thereafter by flow cytometry for AcLDL uptake, E-lectin binding and expression of Tie-2-driven EGFP expression. The histogram represents the relative mean fluorescence of biological triplicate samples +/- 1 S.E. which are expressed relative to that of tumor cells cultured under standard conditions (day 0).
(TIF)

Figure S3 **OVCAR3 TDECs increase tumor vessel density.** EGFP-tagged TDECs were generated from OVCAR3 cells under condition 4 for 5 d, mixed with a 20-fold excess of DsRed-tagged OVCAR3 cells grown under standard conditions and inoculated into the flanks of nude mice as described for Figure 4. Control tumors consisted of the same proportion of EGFP-tagged OVCAR3 cells and DsRed-tagged OVCAR3 cells both grown under standard under conditions. (A) Typical frozen sections of tumors from each group are shown demonstrating a greater contribution of TDECs to the tumor vasculature in the former tumors. (B) Hematoxylin-eosin-stained sections of tumors from each group. Note the greater density of the vasculature from the tumors originating from the inocula containing in vitro-generated TDECs versus those containing control tumor cells. Blood vessels are indicated by black arrows. (C) Graphical depiction of the mean number of tumor blood vessels per field (±SEM) in typical fields of each tumor type. The total number of fields examined as per Figure 4 was 69 for condition 3 tumors and 24 for standard condition tumors. Statistical analysis was performed using a one-tailed Student’s *t* test.
(TIF)
