# Supplementary figures and images for: Rapid In Vitro Derivation of Endothelium Directly From Human Cancer Cells
Source: PLoS One. 2013 Oct 9;8(10):e77675. doi: 10.1371/journal.pone.0077675 (PMC3793940; doi:10.1371/journal.pone.0077675)

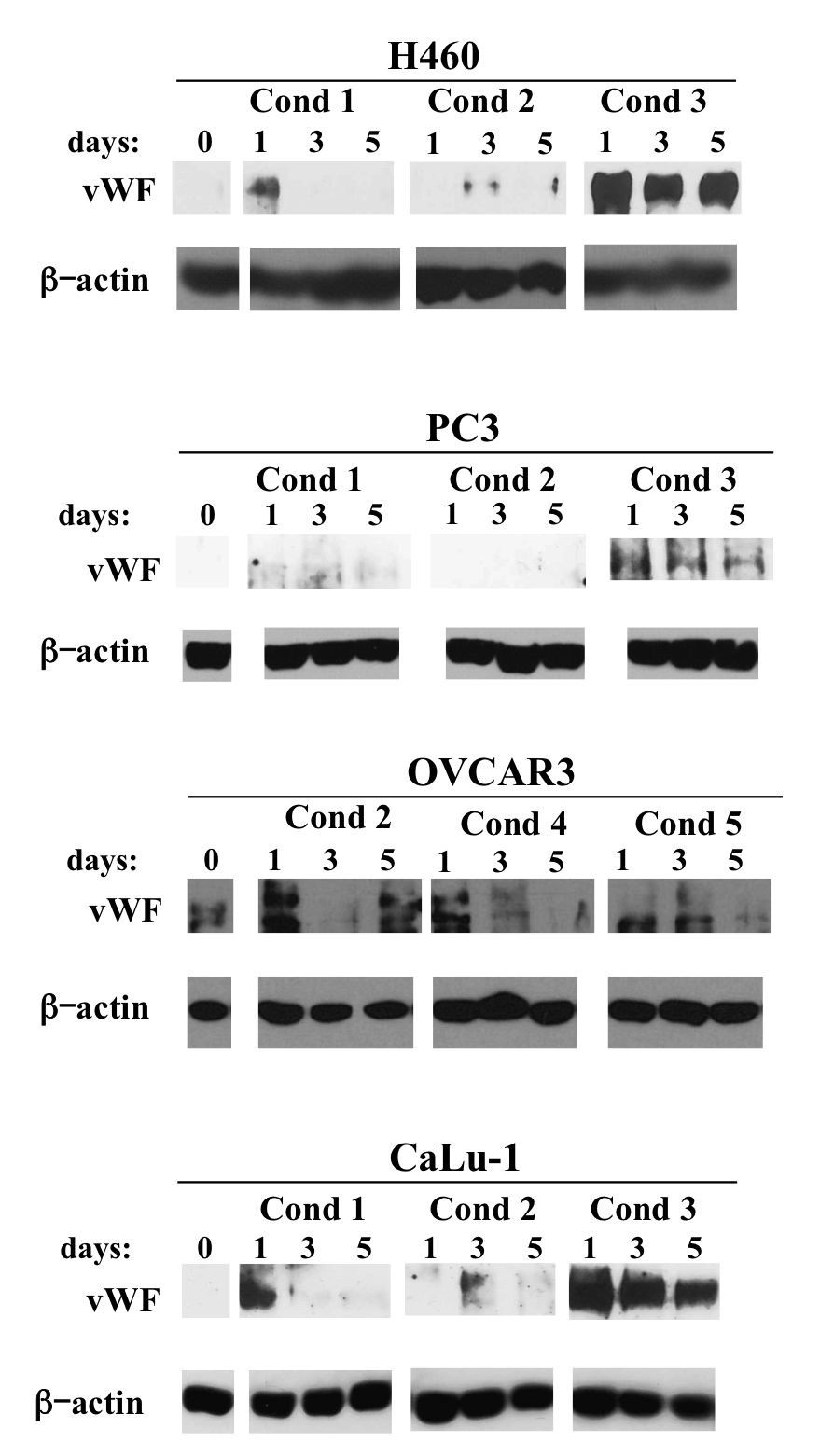

Supplement: Figure S1 — Time course of vWF induction in H460, PC3, OVCAR3 and CaLu1 cells exposed to various conditions. H460, CaLu-1, PC3, and OVCAR cells cultured under the following conditions: “control”, standard growth medium in normoxia; “condition 1”, EC-specific EGM2 medium in normoxia; “condition 2”, standard growth medium in hypoxia (1% O2); “condition 3”, EC-specific EGM2 medium in hypoxia; “condition 4” nutrient-deficient GlutaMax medium in hypoxia; and “condition 5”, nutrient-deficient GlutaMax medium in normoxia. After 1, 3, and 5 days, cells were harvested, whole cell extracts were prepared and equivalent amounts of protein were subjected to immunoblotting for von Willebrands Factor (vWF) and beta-actin, as previously described [24,25]. (TIF) [file pone.0077675.s001.tif]

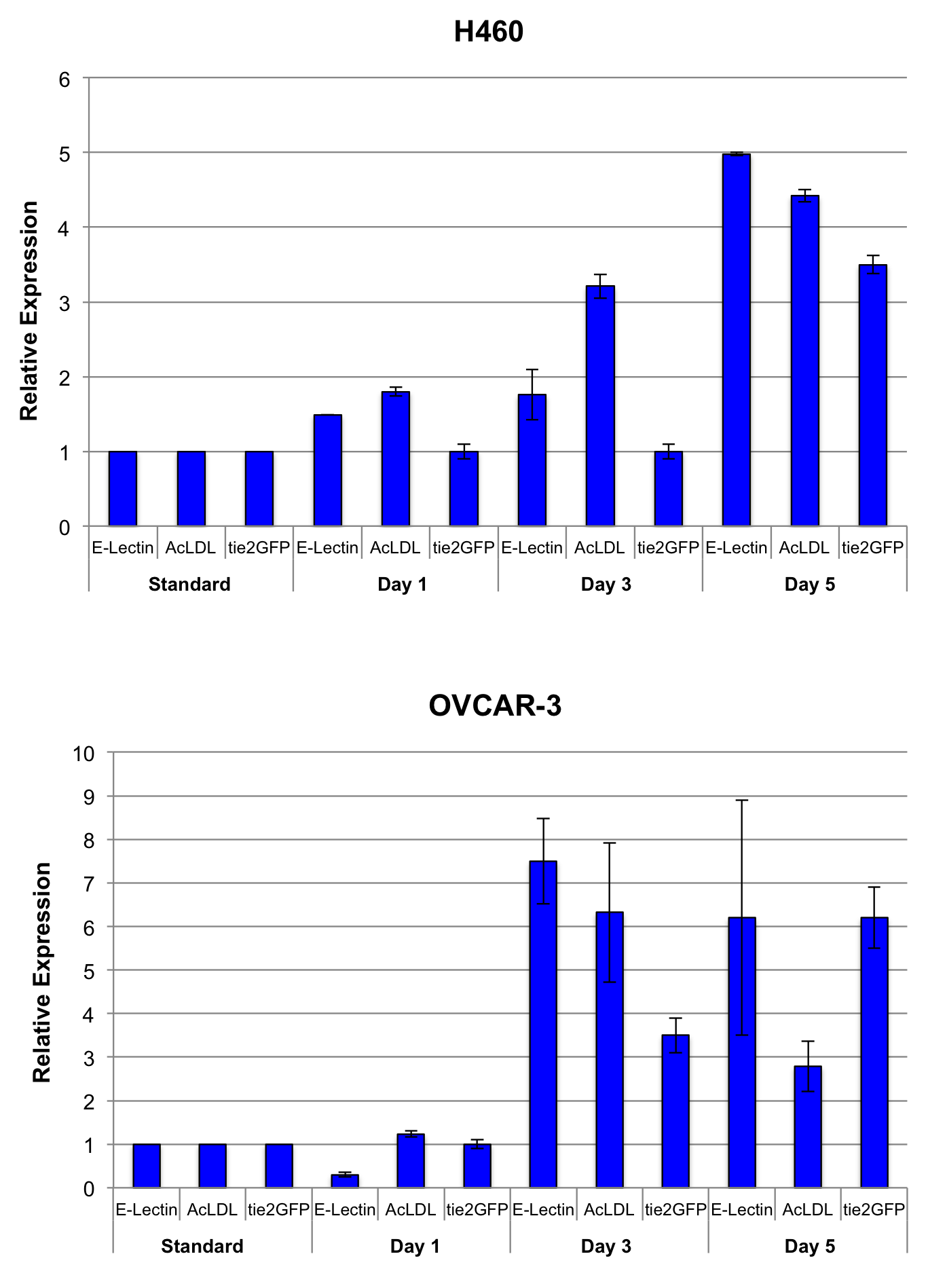

Supplement: Figure S2 — Time course of tumor cellTDEC transition. H460, OVCAR3 or H460-Tie2-EGFP and OVCAR3-Tie2-EGFP cells were subjected to EC-promoting conditions and then assayed periodically thereafter by flow cytometry for AcLDL uptake, E-lectin binding and expression of Tie-2-driven EGFP expression. The histogram represents the relative mean fluorescence of biological triplicate samples +/- 1 S.E. which are expressed relative to that of tumor cells cultured under standard conditions (day 0). (TIF) [file pone.0077675.s002.tif]

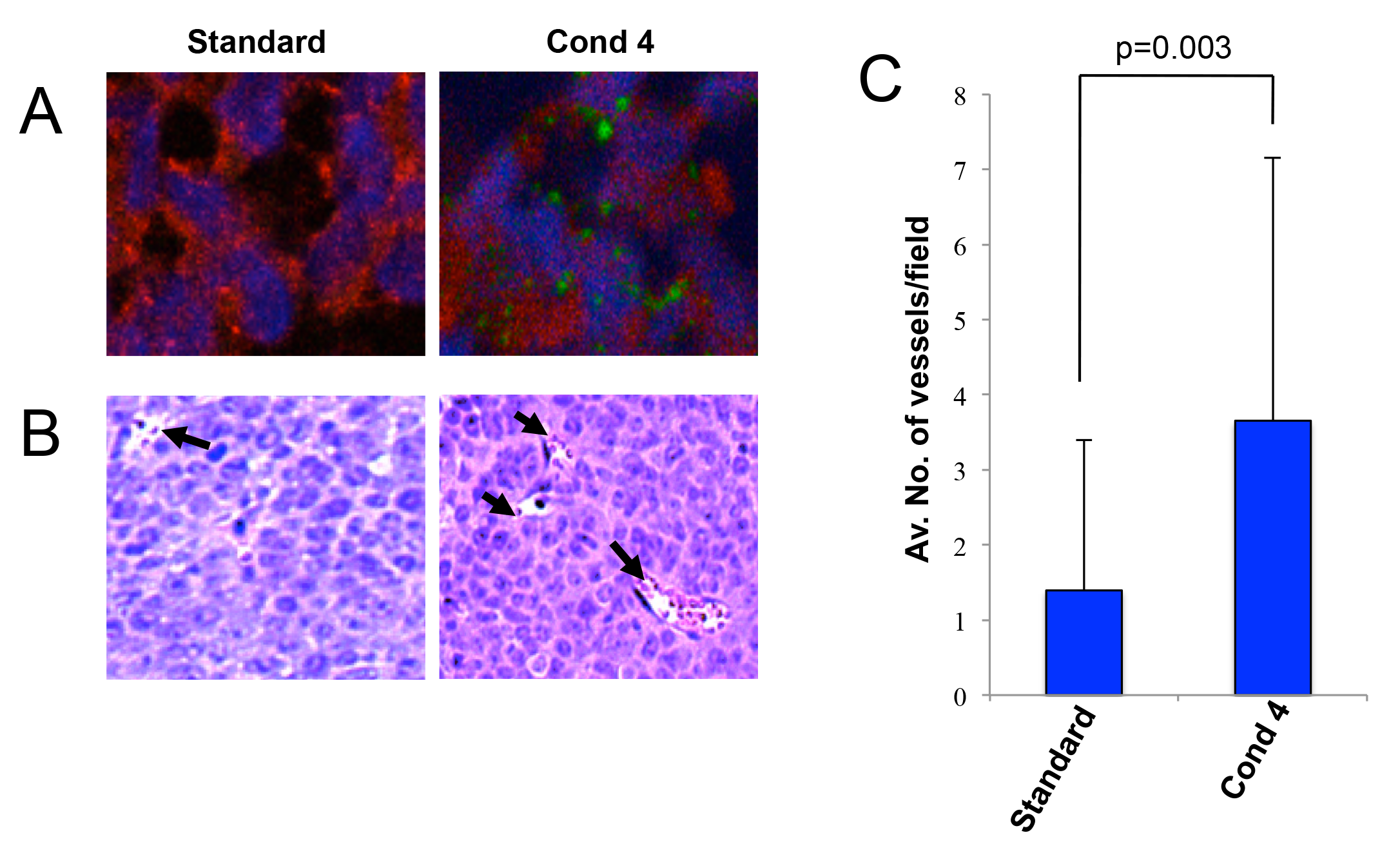

Supplement: Figure S3 — OVCAR3 TDECs increase tumor vessel density. EGFP-tagged TDECs were generated from OVCAR3 cells under condition 4 for 5 d, mixed with a 20-fold excess of DsRed-tagged OVCAR3 cells grown under standard conditions and inoculated into the flanks of nude mice as described for Figure 4. Control tumors consisted of the same proportion of EGFP-tagged OVCAR3 cells and DsRed-tagged OVCAR3 cells both grown under standard under conditions. (A) Typical frozen sections of tumors from each group are shown demonstrating a greater contribution of TDECs to the tumor vasculature in the former tumors. (B) Hematoxylin-eosin-stained sections of tumors from each group. Note the greater density of the vasculature from the tumors originating from the inocula containing in vitro-generated TDECs versus those containing control tumor cells. Blood vessels are indicated by black arrows. (C) Graphical depiction of the mean number of tumor blood vessels per field (±SEM) in typical fields of each tumor type. The total number of fields examined as per Figure 4 was 69 for condition 3 tumors and 24 for standard condition tumors. Statistical analysis was performed using a one-tailed Student’s t test. (TIF) [file pone.0077675.s003.tif]
